# MCM10 overexpression implicates adverse prognosis in urothelial carcinoma

**DOI:** 10.18632/oncotarget.12795

**Published:** 2016-10-21

**Authors:** Wei-Ming Li, Chun-Nung Huang, Hung-Lung Ke, Ching-Chia Li, Yu-Ching Wei, Hsin-Chih Yeh, Lin-Li Chang, Chun-Hsiung Huang, Peir-In Liang, Bi-Wen Yeh, Ti-Chun Chan, Chien-Feng Li, Wen-Jeng Wu

**Affiliations:** ^1^ Graduate Institute of Medicine, College of Medicine, Kaohsiung Medical University, Kaohsiung, Taiwan; ^2^ Department of Urology, Kaohsiung Medical University Hospital, Kaohsiung, Taiwan; ^3^ Department of Urology, School of Medicine, College of Medicine, Kaohsiung Medical University, Kaohsiung, Taiwan; ^4^ Department of Urology, Ministry of Health and Welfare Pingtung Hospital, Pingtung, Taiwan; ^5^ Department of Urology, Kaohsiung Municipal Ta-Tung Hospital, Kaohsiung, Taiwan; ^6^ Department of Pathology, Kaohsiung Municipal Ta-Tung Hospital, Kaohsiung, Taiwan; ^7^ Department of Microbiology, School of Medicine, College of Medicine, Kaohsiung Medical University, Kaohsiung, Taiwan; ^8^ Department of Pathology, Kaohsiung Medical University Hospital, Kaohsiung, Taiwan; ^9^ Department of Pathology, Chi-Mei Medical Center, Tainan, Taiwan; ^10^ Departments of Biotechnology, Southern Taiwan University of Science and Technology, Tainan, Taiwan; ^11^ National Cancer Research Institute, National Health Research Institutes, Tainan, Taiwan; ^12^ Institute of Clinical Medicine, Kaohsiung Medical University, Kaohsiung, Taiwan; ^13^ Department of Internal Medicine and Cancer Center, Kaohsiung Medical University Hospital, Kaohsiung Medical University, Kaohsiung, Taiwan; ^14^ Center for Infectious Disease and Cancer Research, Kaohsiung Medical University, Kaohsiung, Taiwan; ^15^ Center for Stem Cell Research, Kaohsiung Medical University, Kaohsiung, Taiwan; ^16^ Institute of Medical Science and Technology, National Sun Yat-sen University, Kaohsiung, Taiwan

**Keywords:** urothelial carcinoma, transcriptome, MCM10, prognosis

## Abstract

Urothelial carcinoma (UC) occurs in the upper urinary tract (UTUC) and the urinary bladder (UBUC). The molecular pathogenesis of UC has not been fully elucidated. Through data mining of a published transcriptome of UBUC (GSE31684), we identified *Minichromosome Maintenance Complex Component 2* (*MCM2*) and *MCM10* as the two most significantly upregulated genes in UC progression among the *MCM* gene family, the key factors for the initiation of DNA replication. To validate the clinical significance of MCM2 and MCM10, immunohistochemistry, evaluated by H-score, was used in a pilot study of 50 UTUC and 50 UBUC samples. Only a high expression level of MCM10 predicted worse disease-specific survival (DSS) and inferior metastasis-free survival (MeFS) for both UTUC and UBUC. Correspondingly, evaluation of *MCM10* mRNA expression in 36 UTUCs and 30 UBUCs showed significantly upregulated levels in high stage UC, suggesting its role in tumor progression. Evaluation of 340 UTUC and 296 UBUC tissue samples, respectively, demonstrated that high MCM10 immunoexpression was significantly associated with advanced primary tumors, nodal status, and the presence of vascular invasion in both groups of UCs. In multivariate Cox regression analyses, adjusted for standard clinicopathological features, MCM10 overexpression was independently associated with DSS (UTUC hazard ratio [HR]=2.401, P = 0.013; UBUC HR=4.323, P=0.001) and with MeFS (UTUC HR=3.294, P<0.001; UBUC HR=1.972, P=0.015). *In vitro*, knockdown of *MCM10* gene significantly suppressed cell proliferation in both J82 and TCCSUP cells. In conclusion, MCM10 overexpression was associated with unfavorable clinicopathological characteristics and independent negative prognostic effects, justifying its potential theranostic value in UC.

## INTRODUCTION

Urothelial carcinoma (UC) is the most common pathological type of urinary tract cancer. Upper urinary tract urothelial carcinoma (UTUC) comprises only 5% of all urothelial tumors in Western countries [1], but its incidence is high in Taiwan, with a ratio 3.08:6.72, UTUC to urinary bladder urothelial carcinoma (UBUC) [2]. Despite advances in surgical technique and improved understanding of UC carcinogenesis [3–7], 5-year survival rates for patients remain suboptimal [8, 9]. Tumor stage, grade, lymph node status, lymphovascular invasion, tumor architecture, and an infiltrative growth pattern were pathological variables identified as independent prognostic factors of recurrent UC [8, 9]. However, limitations remain for these pathological characteristics to predict the outcome of patients with UC. Accordingly, additional biomarkers are needed to detect and predict outcomes for personalized treatment of UC.

Cell proliferation, a hallmark of cancer development, is required to expand populations of cells with molecular alterations [10, 11]. Some cell proliferation markers are effective in cancer prognostication. The most commonly used markers, such as Ki-67 and proliferating cell nuclear antigen (PCNA) [12, 13], identify malignant cancer cells because their expression coincides with DNA synthesis. Nevertheless, the utility of these markers may not be ideal. Because they do not recognize cells in the G1 phase of the cell cycle- an extended stage in many proliferating cancer cells.

The minichromosome maintenance (MCM) proteins are expressed when G0 cells enter the G1 phase, before they engage in active DNA synthesis [14]. Mcm10 functions in replication licensing at origins by converting an inactive double hexamer of Mcm2-7 into two CMG (Cdc45–MCM–GINS) complexes around dsDNA near replication forks [15]. MCM proteins can be detected in abnormal precursor malignant cells before malignant transformation is completed. Previous studies have reported that MCM protein are prospective prognosis markers in prostate [16] and lung cancers [17].

With the aim of identifying members of the MCM class that are relevant for UC, we searched for *MCM* family expression using a published transcription profiling database in Gene Expression Omnibus (GEO). We found that MCM10 was highly expressed in advanced stage human UBUCs, suggesting a role for this protein in cancer progression. Here, we investigate the association between MCM10 expression and UC prognosis.

## RESULTS

### *MCM2* and *MCM10* were significantly upregulated in UC

**Figure 1:**
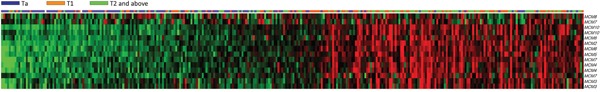
Analysis of transcriptome dataset in urothelial carcinoma from a published transcriptomic dataset (GSE32894) Clustering analysis of genes focusing on the *MCM* gene family revealed that *MCM10* is the most significantly up-regulated gene associated with increments in the pT status, followed by *MCM2*, prompting us to further validate their significance in our pilot batch of cases. Tissue specimens from tumors with different pT statuses are indicated at the top of the heatmap, and expression levels of up-regulated and down-regulated genes are represented using a brightness spectrum of red and green, respectively. Cases with unaltered mRNA transcriptional levels are coded black.

Several *MCM* gene transcripts were found to be up- or downregulated in UC (Table 1 and Figure 1). Only *MCM2* and *MCM10* were identified as significantly upregulated, showing a log_2_ ratio >1 when comparing deep and non-invasive lesions. Of note, only *MCM10* revealed a significant stepwise upregulation from pTa to pT1 and pT1 to pT2-4 (log_2_ ratio of 0.8053 and 0.3815, respectively). Because *MCM2* and *MCM10* were the two most significantly upregulated genes, we validated the significance of the elevated expression in a pilot batch of samples.

**Table 1:** Summary of differentially expressed genes of MCM family and showed stepwise alterations during cancer progression in the transcriptome of urothelial carcinoma of urinary bladder (GSE32894)

Probe	Comparing T2-4 to Ta		Comparing T1 to Ta		Comparing T2-4 to T1		Gene Symbol	Biological Process	Molecular Function
	log ratio	p-value	log ratio	p-value	log ratio	p-value			
ILMN_1663195	0.6328	<0.0001	0.5425	<0.0001	0.0904	0.2971	*MCM7*	DNA replication, DNA replication initiation, cell cycle, regulation of phosphorylation, regulation of transcription; DNA-dependent, response to DNA damage stimulus, transcription	ATP binding, DNA binding, nucleoside-triphosphatase activity, nucleotide binding, protein binding
ILMN_1681503	1.0003	<0.0001	0.8447	<0.0001	0.1556	0.1331	*MCM2*	DNA replication, DNA replication initiation, DNA unwinding during replication, cell cycle, nucleosome assembly, regulation of transcription; DNA-dependent, transcription	ATP binding, DNA binding, DNA replication origin binding, metal ion binding, nucleotide binding, protein binding, zinc ion binding
ILMN_1704702	0.447	<0.0001	0.3643	<0.0001	0.0828	0.1149	*MCM7*	DNA replication, DNA replication initiation, cell cycle, regulation of phosphorylation, regulation of transcription; DNA-dependent, response to DNA damage stimulus, transcription	ATP binding, DNA binding, nucleoside-triphosphatase activity, nucleotide binding, protein binding
ILMN_1737205	0.5055	<0.0001	0.446	<0.0001	0.0595	0.5164	*MCM4*	DNA replication, DNA replication initiation, DNA unwinding during replication, regulation of transcription; DNA-dependent, transcription	ATP binding, DNA binding, DNA helicase activity, hydrolase activity, nucleoside-triphosphatase activity, nucleotide binding, protein binding, single-stranded DNA binding
ILMN_1798581	0.437	0.0002	0.4273	0.0002	0.0097	0.9127	*MCM8*	DNA replication, cell cycle, regulation of transcription; DNA-dependent, transcription	ATP binding, DNA binding, nucleoside-triphosphatase activity, nucleotide binding
ILMN_1798654	0.7221	<0.0001	0.583	<.00010	0.1391	0.1232	*MCM6*	DNA replication, DNA replication initiation, DNA unwinding during replication, cell cycle, regulation of transcription; DNA-dependent, transcription	ATP binding, DNA binding, DNA helicase activity, identical protein binding, nucleotide binding, single-stranded DNA binding
ILMN_1800654	0.1068	0.0025	0.1454	0.0001	−0.0386	0.238	*MCM7*	DNA replication, DNA replication initiation, cell cycle, regulation of phosphorylation, regulation of transcription; DNA-dependent, response to DNA damage stimulus, transcription	ATP binding, DNA binding, nucleoside-triphosphatase activity, nucleotide binding, protein binding
ILMN_1806818	0.204	0.039	0.4568	<0.0001	−0.2529	0.0082	*MCM3*	DNA replication, DNA replication initiation, cell cycle, regulation of transcription; DNA-dependent, transcription	ATP binding, DNA binding, nucleoside-triphosphatase activity, nucleotide binding, protein binding
ILMN_1815169	0.5307	<0.0001	0.5146	<0.0001	0.016	0.8954	*MCM5*	DNA replication, DNA replication initiation, cell division, regulation of transcription; DNA-dependent, transcription	ATP binding, DNA binding, nucleotide binding, protein binding
ILMN_2224143	0.1157	0.1739	0.2752	0.0009	−0.1595	0.052	*MCM3*	DNA replication, DNA replication initiation, cell cycle, regulation of transcription; DNA-dependent, transcription	ATP binding, DNA binding, nucleoside-triphosphatase activity, nucleotide binding, protein binding
ILMN_2407124	0.4594	<0.0001	0.3002	<0.0001	0.1592	0.1378	*MCM8*	DNA replication, cell cycle, regulation of transcription; DNA-dependent, transcription	ATP binding, DNA binding, nucleoside-triphosphatase activity, nucleotide binding
ILMN_2412860	0.4652	<0.0001	0.4671	<0.0001	−0.0019	0.9842	*MCM4*	DNA replication, DNA replication initiation, DNA unwinding during replication, regulation of transcription; DNA-dependent, transcription	ATP binding, DNA binding, DNA helicase activity, hydrolase activity, nucleoside-triphosphatase activity, nucleotide binding, protein binding, single-stranded DNA binding
ILMN_2413898	1.1868	<0.0001	0.8053	<0.0001	0.3815	<0.0001	*MCM10*		
ILMN_2413899	0.5286	<0.0001	0.3248	<0.0001	0.2038	0.0001	*MCM10*		

### MCM10 expression was significantly associated with tumor aggressiveness

**Figure 2:**
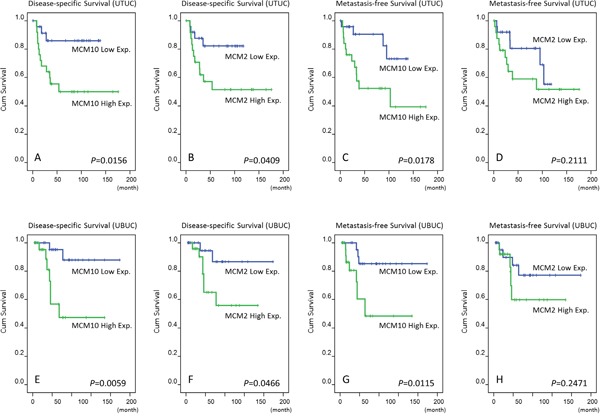
Validation using immunohistochemistry for our pilot batch of 50 upper urinary tract urothelial carcinomas (UTUC, A, B, C, D) and urinary bladder urothelial carcinomas (UBUC, E, F, G, H) MCM10 high expression significantly predicted inferior disease-specific survival (DSS) and metastasis-free survival (MeFS) for both UTUCs and UBUCs, while MCM2 is predictive only for DSS.

In the initial study, both MCM2 and MCM10 overexpression were significantly associated with primary tumor status in UTUC (*P*=0.048 and 0.004, respectively) and UBUC (*P*=0.005 and 0.004, respectively) (Supplementary Table S1). Interestingly, both MCM2 and MCM10 overexpression predicated worse DSS for UTUC (*P*=0.0409 and 0.0156, respectively) and UBUC (*P*=0.0466 and 0.0059, respectively). However, only MCM10 predicated inferior MeFS for both UTUC and UBUC (P=0.0178 and 0.0115, respectively; Figure 2 and Supplementary Table S2) and was an independent prognosticator after multivariate analysis (Supplementary Table S3)

### *MCM10* mRNA expression increases with higher pT stages in both UTUC and UBUC

**Figure 3:**
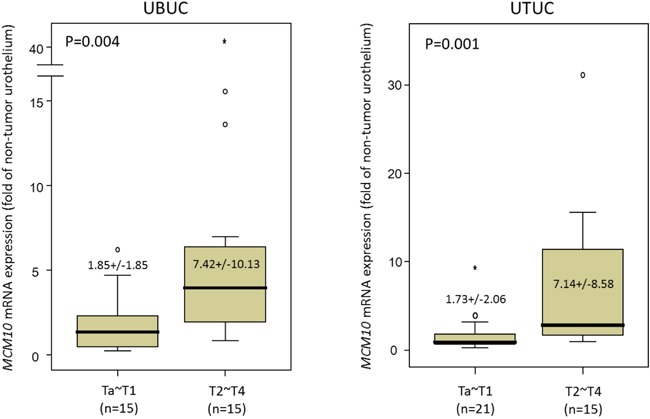
QuantiGene assay *MCM10* mRNA expression was significantly increased in both UBUCs (*left* panel) and UTUCs (*right* panel) with advanced primary pT status. (p=0.004 and p=0.001, respectively).

We evaluated *MCM10* transcript expression in each of 35 UTUC and 30 UBUC samples. *MCM10* mRNA expression was significantly upregulated in higher stage tumors in both UTUC (P=0.001) and UBUC (P=0.004) tissue, suggesting its role in cancer progression (Figure 3A and 3B).

### Clinicopathological features of UTUC

Table 2 shows the clinicopathological parameters of the UTUC patients. There was no gender preference. The median age at diagnosis was 68 years (range, 34 to 87 years). Sixty-two (18.2%) patients had multifocal tumors. Forty-nine (14.4%) patients suffered from tumors involving both the renal pelvis and ureter. Most UTUCs (n=284, 83.5%) were of high tumor grade. Around half the patients (159, 46.8%) presented with muscle invasive disease. Lymph node metastasis was detected in 28 (8.2%) patients.

**Table 2:** Correlations between MCM10 expression and other important clinicopathological parameters in urothelial carcinomas

Parameter	Category	Upper Urinary Tract Urothelial Carcinoma				Urinary Bladder Urothelial Carcinoma			
		Case No.	MCM10 Expression		p-value	Case No.	MCM10 Expression		p-value
			Low	High			Low	High	
Gender	Male	158	85	73	0.192	216	111	105	0.376
	Female	182	95	97		79	36	43	
Age (years)	< 65	138	68	70	0.825	121	65	56	0.265
	≥ 65	202	102	100		174	82	92	
Tumor location	Renal pelvis	141	69	72	0.909	-	-	-	-
	Ureter	150	77	73		-	-	-	-
	Renal pelvis & ureter	49	24	25		-	-	-	-
Multifocality	Single	278	141	137	0.574	-	-	-	-
	Multifocal	62	29	33		-	-	-	-
Primary tumor (T)	Ta	89	57	32	<0.001*	84	52	32	<0.001*
	T1	92	58	34		88	53	35	
	T2-T4	159	55	104		123	42	81	
Nodal metastasis	Negative (N0)	312	164	148	0.002*	266	140	126	0.004*
	Positive (N1-N2)	28	6	22		29	7	22	
Histological grade	Low grade	56	42	14	<0.001*	56	38	18	0.003*
	High grade	284	128	156		239	109	130	
Vascular invasion	Absent	234	140	94	<0.001*	246	133	113	0.001*
	Present	106	30	76		49	14	35	
Perineural invasion	Absent	321	168	153	<0.001*	275	142	133	0.021*
	Present	19	2	17		20	5	15	
Mitotic rate (per 10 high power fields)	< 10	173	125	48	<0.001*	139	85	54	<0.001*
	>= 10	167	45	122		156	62	94	

* Statistically significant.

### Clinicopathological features of UBUC

As summarized in Table 2, most UBUC patients were male (n=216, 73.2%) and elderly (more than 65 years; n=214, 72.5%). Most UBUC tumors (n=239, 81%) had a high histological grade. Muscle-invasive disease was diagnosed in 123 (41.7%) patients. Of these, 29 (23.6%) patients had nodal metastases.

### Correlation of MCM10 expression with clinicopathological features in UC

**Figure 4:**
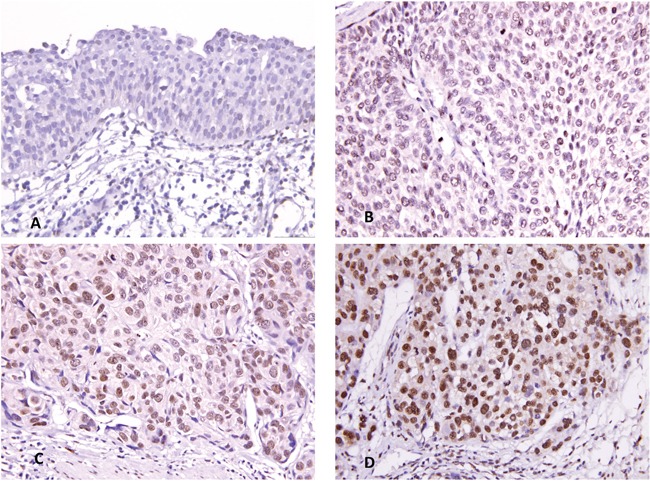
MCM10 immunostaining Representative sections of non-tumor urothelium **A.**, non-invasive urothelial carcinoma **B.**, superficially invasive urothelial carcinoma **C.**, and high-stage infiltrating urothelial carcinoma **D.** exhibit a stepwise increment.

Because *MCM10* was identified as the most significantly upregulated gene in *MCM* family, we further explored the significance of its expression in a large cohort of cases using immunohistochemistry. Increased MCM10 expression increased with increasing pT stage (Figure 4). In the 635 cases, MCM10 showed variable nuclear expression in both UTUC and UBUC with median H-scores of 160 (range, 100-380) and 165 (range, 100-370), respectively. After the tumors were dichotomized into those with low and high MCM10 expression (Table 2), high MCM10 was significantly associated with increased tumor stage (both UTUC and UBUCP<0.001), higher histological grade (UTUC P<0.001; UBUC, *P*=0.003), lymph node metastasis (UTUC, P=0.002; UBUC, P=0.004), vascular invasion (UTUC, P<0.001; UBUC, P=0.001), perineurial invasion (UTUC, P<0.001; UBUC, P=0.021) and frequent mitosis (both UTUC and UBUCP<0.001). These findings suggested a potential role of MCM10 in the progression of UCs.

### Patient outcome for UTUC

**Figure 5:**
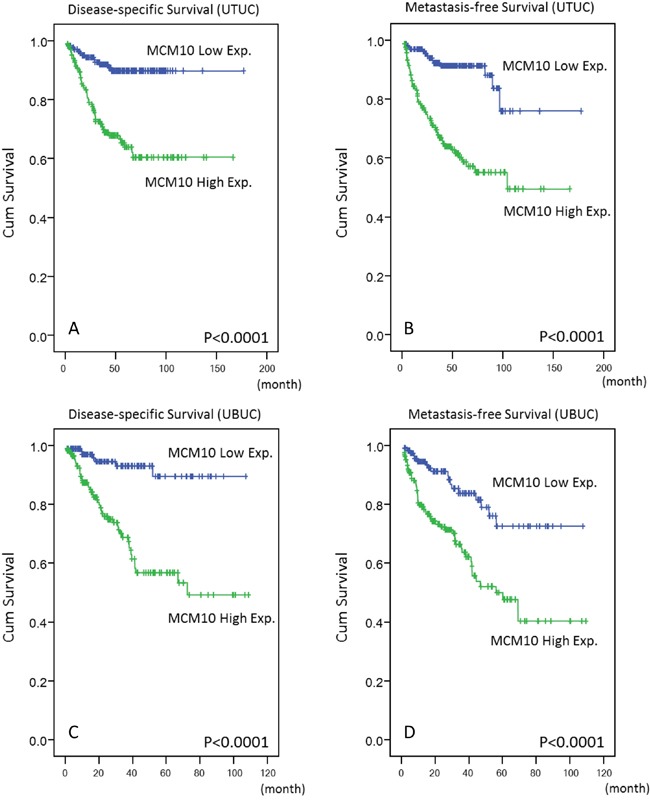
Kaplan-Meier plots These plots show the prognostic significance of MCM10 expression for DSS and MeFS of UTUC **A & B.** and UBUC **C & D.**

Follow-up information was available for all UTUC patients for 1 to 176 months (median, 38 months). Table 3 summarizes the association between patient outcomes and important clinicopathological parameters. Multivariate analysis revealed a number of features that predicted poor DSS, including multifocality (P=0.018), histological grade (P=0.020), lymph node metastasis (P<0.001), and perineurial invasion (P=0.002). Similar results were observed for MeFS. Vascular invasion in UTUC was also independently associated with worse MeFS (P=0.009). More importantly, patients with high MCM10 expression had significantly worse DSS and MeFS in both univariate (P<0.0001, Figure 5A and 5B) and multivariate analyses (P=0.013 and P<0.001, respectively).

**Table 3:** Correlations between MCM10 expression and other important clinicopathological parameters in urothelial carcinomas

Parameter	Category	Case No.	Disease-specific Survival					Metastasis-free Survival				
			Univariate analysis		Multivariate analysis			Univariate analysis		Multivariate analysis		
			No. of event	p-value	R.R.	95% C.I.	p-value	No. of event	p-value	R.R.	95% C.I.	p-value
**Gender**	Male	158	28	0.8286	-	-	-	32	0.7904	-	-	-
	Female	182	33		-	-	-	38		-	-	-
**Age (years)**	< 65	138	26	0.9943	-	-	-	30	0.8470	-	-	-
	≥ 65	202	35		-	-	-	40		-	-	-
**Tumor side**	Right	177	34	0.7366	-	-	-	38	0.3074	-	-	-
	Left	154	26		-	-	-	32		-	-	-
	Bilateral	9	1		-	-	-	0		-	-	-
**Tumor location**	Renal pelvis	141	24	0.0079*	1	**-**	0.706	31	0.0659	-	-	-
	Ureter	150	22		0.820	0.440-1.526		25		-	-	-
	Renal pelvis & ureter	49	15		1.361	0.377-4.913		14		-	-	-
**Multifocality**	Single	273	48	0.0026*	1	-	0.018*	52	0.0127*	1	**-**	0.007*
	Multifocal	62	18		2.530	1.173-5.455		18		2.127	**1.224-3.697**	
**Primary tumor (T)**	Ta	89	2	<0.0001*	1	-	0.063	4	<0.0001*	1	-	0.189
	T1	92	9		3.337	0.708-15.718		15		2.792	0.901-8.650	
	T2-T4	159	50		5.546	1.231-24.979		51		2.684	0.853-8.440	
**Nodal metastasis**	Negative (N0)	312	42	<0.0001*	1	-	<0.001*	55	<0.0001*	1	-	0.001*
	Positive (N1-N2)	28	19		5.077	2.740-9.450		15		2.962	1.603-5.472	
**Histological grade**	Low grade	56	4	0.0215*	1	-	0.020*	3	0.0027*	1	-	0.014*
	High grade	284	57		3.729	1.234-11.272		67		4.550	1.351-15.301	
**Vascular invasion**	Absent	234	24	<0.0001*	1	-	0.130	26	<0.0001*	1	-	0.009*
	Present	106	37		1.579	0.874-2.853		44		2.226	1.223-4.053	
**Perineural invasion**	Absent	321	50	<0.0001*	1	**-**	0.002*	61	<0.0001*	1	-	0.041*
	Present	19	11		3.248	1.537-6.864		9		2.181	1.032-4.609	
**Mitotic rate (per 10 high power fields)**	< 10	173	27	0.167	-	-	-	30	**0.0823**	-	-	-
	>= 10	167	34		-	-	-	40		-	-	-
**MCM10 expression**	Low	170	12	<0.0001*	1	-	0.013*	13	<0.0001*	1	-	<0.001*
	High	170	49		2.401	1.201-4.800		57		3.294	1.704-6.367	

* Statistically significant.

### Survival analysis for UBUC

The median follow-up time was 23.1 months (range, 1 to 109 months) in UBUC patients. As shown in Table 4, pT stage and mitotic rate significantly predicted both inferior DSS (P<0.001 and P=0.024, respectively) and MeFS (*P*=0.015 and *P*=0.019, respectively) in multivariate analysis. Of note, MCM10 overexpression also predicted inferior DSS and MeFS after both univariate (P<0.0001, Figure 5C and 5D) and multivariate analyses (P=0.001 and P=0.015, respectively).

**Table 4:** Univariate log-rank and multivariate analyses for Disease-specific and Metastasis-free Survivals in urinary bladder urothelial carcinoma

Parameter	Category	Case No.	Disease-specific Survival					Metastasis-free Survival				
			Univariate analysis		Multivariate analysis			Univariate analysis		Multivariate analysis		
			No. of event	p-value	R.R.	95% C.I.	p-value	No. of event	p-value	R.R.	95% C.I.	p-value
**Gender**	Male	216	41	0.4446	-	-	-	60	0.2720	-	-	-
	Female	79	11		-	-	-	16		-	-	-
**Age (years)**	< 65	121	17	0.1136	-	-	-	31	0.6875	-	-	-
	≥ 65	174	35		-	-	-	45		-	-	-
**Primary tumor (T)**	Ta	84	1	<0.0001*	1	-	<0.001*	4	<0.0001*	1	-	0.015*
	T1	88	9		7.441	0.790-70.108		23		5.546	1.597-19.252	
	T2-T4	123	42		29.581	3.184-274.822		49		8.230	2.352-28.798	
**Nodal metastasis**	Negative (N0)	266	41	0.0002*	1	-	0.761	61	<0.0001*	1	-	0.100
	Positive (N1-N2)	29	11		1.114	0.554-2.239		15		1.670	0.906-3.097	
**Histological grade**	Low grade	56	2	0.0013*	1	-	0.639	5	0.0007*	1	-	0.938
	High grade	239	50		0.684	0.140-3.345		71		1.043	0.362-3.002	
**Vascular invasion**	Absent	246	37	0.0024*	1	-	0.135	54	0.0001*	1	-	0.812
	Present	49	15		0.585	0.289-1.182		22		0.930	0.511-1.692	
**Perineural invasion**	Absent	275	44	0.0001*	1	-	0.066	66	0.0007*	1	-	0.206
	Present	20	8		2.233	0.947-5.262		10		1.625	0.766-3.450	
**Mitotic rate (per 10 high power fields)**	< 10	139	12	<0.0001*	1	-	0.024*	23	<0.0001*	1	-	0.019*
	>= 10	156	40		2.145	1.104-4.170		53		1.843	1.105-3.075	
**MCM10 expression**	Low	147	10	<0.0001*	1	-	0.001*	23	<0.0001*	1	-	0.015*
	High	148	42		4.323	1.797-10.399		53		1.972	1.139-3.416	

* Statistically significant.

### MCM10 expression promotes growth of urothelial cells *in vitro*

**Figure 6:**
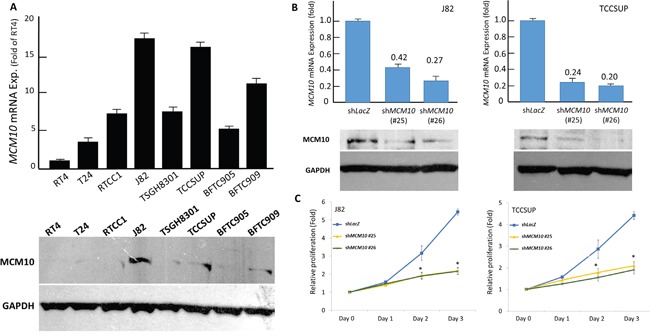
MCM10 expression promotes growth of UC cells *in vitro* **A.** Compared to RT4 cells, endogenous *MCM*10 mRNA (*upper*) and protein (*lower*) expression is higher in J82 and TCCSUP cell lines. **B.** The two cell lines with high endogenous MCM10 expression are stably silenced against MCM10 expression by a lentiviral vector bearing one of the two clones of MCM10 shRNA with different sequences for both J82 (*left* panel) and TCCSUP (*right* panel) cells. The efficiency of RNA silencing is confirmed by both quantitative RT-PCR (*upper* row) and western blotting (*lower* row) assays. The *shLacZ* plasmid, *POLR2A* transcript, and GADPH protein are utilized as controls in RNA interference, quantitative RT-PCR, and western blotting assays, respectively. **C.** Using an ELISA-based, colorimetric assay to assess the rate of BrdU uptake, cell proliferation is significantly reduced in stable *MCM10*-knockdown J82 (*left*) and TCCSUP (*right*) cell lines, compared to the corresponding *shLacZ* controls. (*, *P*<0.05).

To gain insight into the biology, we next characterized the UC cell lines for their endogenous MCM10 expression. Of the eight UC cell lines tested, the endogenous expression levels of *MCM10* mRNA and protein were higher in J82 and TCCSUP cells using RT4 cells as the baseline reference (Figure 6A). We thus employed RNA interference to decipher the functional effects of MCM10 overexpression, and remarkable silencing of MCM10 expression was achieved in selected stable clones of J82 (Figure 6B, *left*) and TCCSUP cells (Figure 6B, *right*). Compared with their *shLacZ* controls, the BrdU incorporation rates in both stable *MCM10*-silenced J82 and TCCSUP cells were significantly attenuated (Figure 6C). This finding indicated the growth-promoting role of MCM10.

## DISCUSSION

Currently, few effective biomarkers for early prognostication of UC exist. In this study, we identified a molecule that could provide additional information for designing further treatment plans and that goes beyond traditional TNM staging or tumor grade. Because cell proliferation is a hallmark of cancer [10, 11], we focused on *MCM* genes, key factors for initiation of DNA replication. Through data mining, we identified two potential prognosticators, *MCM2* and *MCM10*, and performed a pilot study to validate the role of these genes in UC. Only MCM10 overexpression significantly predicted worse DSS and inferior MeFS for both UTUC and UBUC. We further confirmed the clinical significance of MCM10 expression in well-characterized cohorts of 340UTUCs and 296 UBUCs. We demonstrated that MCM10 immunoexpression was significantly associated with aggressive pathological features, including advanced primary tumor status, vascular invasion, and nodal metastasis in both groups of UCs. MCM10 overexpression was independently associated with MeFS and DSS. These findings indicate that standard clinicopathological practice could benefit by adding MCM10 status to improve risk stratification for UC.

MCM2–7, a complex of six subunits, is an essential component of the prereplication chromatin assembled at the replication origins during the G1 phase and with the processive helicase at growing forks. In the G1 phase of the cell cycle, Cdc6, Cdt1, and Mcm2-7 are recruited to the replication origins in an origin recognition complex-dependent process to form a prereplicative complex (pre-RC) [18]. Upon entry into S phase, the conversion of pre-RCs into active replication forks initiates DNA replication. This transformation requires the activity of two families of protein kinases, the Cdc7/Dbf4 kinases and the S phase cyclin-dependent kinases (Cdks) [19]. The loaded complex is then activated *in situ* during the S-phase by recruitment of Cdc45 protein and the GINS complex to form the active Cdc45–MCM–GINS (CMG) helicase, which has a variety of regulatory factors assembled around it at nascent DNA replication forks [20]. Subsequently, the formation of the replisome progression complex acts as a eukaryotic replisome. As part of the protein machinery for replication licensing, the MCM complex is necessary for a quiescent cell to re-enter the cell cycle, and its components are even before DNA synthesis or cancer-associated histological changes. Therefore, it might be a good marker for early detection of malignant changes at the cellular level.

The diagnostic value of MCM proteins was first verified when antibodies against MCM5 andKi-67 were compared in cervical squamous intraepithelial lesions [21]. In addition, elevated MCM2 expression in the high growth fraction of B-cell lymphomas has been reported [22]. *MCM2* is also a target gene of miR-31 in suppressing medulloblastoma cell growth [23]. Furthermore, poor patient survival corresponds with increased expression of MCM2 in prostate [24], lung [25] and breast [26] cancer cells. Together these studies suggest that MCM proteins are sensitive and versatile diagnostic markers for early cancer detection and may be promising prognostic markers.

Mcm10, chromatin-associated protein, is a replication initiation protein that physically interacts with members of the MCM2–7 complex [27]. Mcm10 is an evolutionally conserved essential protein that facilitates the phosphorylation of Mcm complex to initiate replication [28]. MCM10 is important for efficient DNA replication origin firing in human cells [29]; it also promotes origin unwinding, and promotes the recruitment of both replication protein A (RPA) and DNA polymerases to the origin [30].

In previous studies, defects in the loading or activation of the Mcm2-7 complex in budding yeast impeded checkpoint activation [31]. One study found that checkpoint activation was defective following the depletion of Mcm10 [32]. A different study reported that there might be an interaction between BRCA2 and MCM10, which may serve as a checkpoint, and may be important for stabilizing the replication fork [33]. Loss of these functions results in severe defects in DNA double-strand break repair and may lead to tumorgenesis. Such findings imply that MCM10 could be important for repair of defective genes. However, most studies show its association with malignant behavior of cancer cells.

We found a significant association between MCM10 expression and pT stage. Other parameters, such as node metastasis, tumor grade, and perineurial invasion were significant prognostic factors in UTUC, even in multivariate analysis. However, the only significant prognosticator in UBUC was increasing MCM10 expression. The more invasive the UC, whether UTUC or UBUC, the higher the MCM10 expression. This is consistent with the idea that more cell cycle activation relates to increased proliferation in cancer cells. In addition, we also found that higher expression of MCM10corresponded with worse outcomes of cancer-specific survival and metastasis risk in both types of UC. Our results provide the first data suggesting that MCM10 expression could provide an early warning for poor outcomes in UC, as it is expressed before histological changes are observable and may indicate the need of a more aggressive treatment plan.

MCM10 is a turnkey of the MCM complex in cancer proliferation, making it a strong target for anticancer therapy. In general, it has been suggested that anti-MCM molecules are potential therapeutics for cancer [34]. To date, several studies have reported that anti-MCM small molecules are potentially effective against a broad spectrum of cancers. Kwon et al. explored the ability of widdrol, which down-regulates MCM proteins, to inhibit growth of human colon adenocarcinoma HT29 cells *in vitro* [35]. Mechanistically, anti-MCM small molecules are most likely to stop the proliferation of cancer cells by blocking replication licensing or inhibiting DNA synthesis; however, recent studies showed that they could also induce apoptosis, specifically in cancer cells [36, 37]. We have identified MCM10 as a marker of urothelial carcinoma progression with prognostic value for both upper and lower urinary tract cancers. Our findings suggest that targeting MCM10 in UC could be an effective treatment strategy that warrants future attention.

## MATERIALS AND METHODS

### Data mining of GEO to identify altered *MCM* transcripts in UC

We performed data mining using GEO (National Center Biotechnology information, Bethesda, MD, USA) and identified one dataset, GSE32894 (http://www.ncbi.nlm.nih.gov/geo/query/acc.cgi?acc=GSE32894), which profiled radical cystectomy specimens from 308 UBUC using an Illumina HT12.0 Array. To analyze the gene expression level, we imported the raw files into the Nexus Expression 3 statistical software (BioDiscovery, EI Segundo, CA, USA). All probe sets were used in the analysis without preselection or filtering. We performed supervised comparative analysis to examine the statistical significance of differentially expressed genes based on primary tumor status (pT) progression. Therefore, we compared differential expression between muscle-invasive, high-stage (pT2-pT4), superficially invasive (pT1), and non-invasive (pTa) UCs to obtain functional profiles focused on MCM proteins. Only genes showing significant differential expression (log_2_ ratio >+/−0.1, p<0.01) were enrolled for initial validation.

### Patients and tumor specimens

The institutional review board (IRB) of the Chi Mei Medical Center (CMMC) approved this study (IRB10302015). We retrieved UC cases from the BioBank of CMMC archives between 1996 and 2004 for immunohistochemical study and survival analysis. For the initial validation, aiming to identify the most significant among the candidate genes, a pilot batch of 50 UBUCs and 50UTUCs were randomly selected. The gene showing the most clinical significance was further evaluated in an independent cohort as previously described [38–40]

### Immunohistochemical staining

As described previously [41, 42], tissue sections of 4-μm thickness were cut onto precoated slides from paraffin-embedded tissue blocks, followed by deparaffinization, rehydration, antigen retrieval, and blockage of endogenous peroxidase. The slides were subsequently incubated with a primary antibody targeting one of two significant candidate genes, MCM2 (1:100, N-19, Santa Cruz) or MCM10 (1:150, H-41, Santa Cruz), for one hour. We used the DAKO ChemMate EnVision Kit (K5001, Carpinteria, CA, USA) to detect primary antibodies. The presence of brown cytoplasmic staining of cancer cells indicated positive immunoreactivity. To ensure the quality of immunostaining, incubation without the primary antibody was used as a negative control.

### Interpretation and scoring of immunohistochemistry

All slides were blindly reviewed by two independent pathologists. Immunoreactivity was scored based on a combination of both the percentage and intensity of positively stained tumor cytoplasm to generate an H-score. This was calculated using the following equation: H-score=ΣPi (i+1), where i is the intensity of stained tumor cells (0 to 3+), and Pi is the percentage of stained tumor cells, varying from 0% to 100% [43].

### QuantiGene (branched DNA) assay to determine *MCM10* transcript level

Gene expression was analyzed using the QuantiGene Multiplex 2.0 assay systems (Affymetrix/Panomics Inc., Santa Clara, CA) per the manufacturer's instructions. In brief, custom probes specifically targeting the *MCM10* transcript were designed by Affymetrix (Santa Clara, USA). Oligonucleotides of probe set were mixed with the lysed paraffin sections, and the mixture was added to an assay well in a 96-well plate coated with capture probe oligonucleotide. Target RNA was captured during an overnight incubation at 55°C. Unbound materials was removed by three-run washes with 300μl of wash buffer followed by subsequent hybridization of DNA amplifier molecules, and followed by three washes after incubation every time. After the final wash, the dioxetane alkaline phosphatase substrate Lumiphos Plus (Lumingen Inc., Southfield, MI, USA) was added to the reaction wells. After a short incubation, luminescent signal was detected using a Luminex 100 microplate luminometer (Luminex, TX, USA). The detected readout of *MCM10* mRNA abundance was further normalized through the expression level of reference *POLR2A* transcript.

### Cell culture

Four UC cell lines including RT4, T24, TCCSUP, and J82 were obtained from ATCC (Manassas, VA 20108, USA). TSGH8301, BFTC-905, and BFTC-909 were purchased from Food Industry Research and Development Institute (Hsinchu, Taiwan). An UTUC cell, RTCC1, was obtained from Professor Lien-Chai Chiang at Kaohsiung Medical University [44]. These cells were grown based on suggested medium and conditions.

### Quantification of *MCM10* transcript

Real-time RT-PCR was performed using an ABI StepOnePlus™ System to measure *MCM10* mRNA abundance using the protocol as previously mentioned [45, 46]. RNeasy Mini kit (Qiagen, Valencia, CA) was used to extract total RNAs from human UC cell lines and stable clones of T24 and J82 cells with lentiviral vectors bearing either *shMCM10* or *shLacZ*. RNAs were further reverse-transcribed using SuperScript™ III First-Strand Synthesis System (Invitrogen, Carlsbad, CA) per the manufacturers' instructions. Real-time PCR assay to quantify the expression level of MCM10 transcript was performed using pre-designed TaqMan assay reagents (*MCM10* Hs00218560_m1, and *POLR2A* Hs01108291_m1 from Applied Biosystems, Foster City, CA). The obtained data were normalized by the expression of *POLR2A* housekeeping transcript. After normalization to *POLR2A*, the relative expression fold of *MCM* transcript was then given by 2^−ΔΔCp^, where ΔΔCT = ΔCT _(UC cells)_- ΔCT _(calibrator)_, ΔCT represented the CT of *MCM10* subtracted from the CT of *POLR2A*, and the calibrator was RT4 cell. Only samples with CT value <32 for *POLR2A* were considered to have acceptable RNA quality and included in the analyses.

### Western blot assays

The western blotting assay was performed based on that of our previous publications [45, 46] to evaluate the endogenous MCM10 expression and the efficiency of MCM10 knockdown in J82 and TCCSUP cell lines. Cell lysates containing 25 μg protein were separated by 4-12% gradient NuPAGE gel (Invitrogen, Carlsbad, CA), transferred onto PVDF membranes (Amersham, Biosciences, Buckinghamshire, UK). After blocking with 5% skimmed milk in TBST buffer at room temperature for 1 h, the membranes were then probed with antibodies at 4°C overnight against MCM10 (1:1000, H-41, Santa Cruz), and GAPDH as a loading control (6C5, 1:10,000, Millipore, Beverly, MA). After incubation with the secondary antibody at room temperature for 1.5 h, proteins were visualized by the chemiluminescence system (Amersham Biosciences).

### RNA interference

To establish stably silenced clones of *MCM10*-amplified J82 and TCCSUP cell lines with the short-hairpin RNAs against MCM10 expression (*shMCM10*), the lentiviral vectors were obtained from Taiwan National RNAi Core Facility, including pLKO.1-*shLacZ* (TRCN0000072223: 5′-TGTTCGCATTAT CCGAACCAT-3′) and pLKO.1-*shMCM10* (TRCN0000245425: 5′-TCATCCTCAGAAGG TCTTAAT-3′; TRCN0000245426: 5′-GACGGCGACGG TGAATCTTAT-3′). Viruses were produced by transfecting HEK293 cells with the above three vectors using Lipofectamine 2000 as previously described [45, 47]. For viral infection, 3×10^6^J82 or TCCSUP cells were incubated with 8 ml lentivirus in the presence of polybrene, followed by puromycin selection for stable clones of lentivirus-transduced cells.

### Bromodeoxyuridine (BrdU) assay to assess cell proliferation

Cell proliferation was assessed using an enzyme-linked immunosorbent assay-based and colorimetric BrdU assay (Roche Diagnostics) as previously described [48, 49]. MCM10-knockdown or *shLacZ* control J82 and TCCSUP cells were plated into a 96-well plate at density of 3000 cells per well, and cell proliferation was evaluated at 24, 48, and 72 h. After incubation with BrdU for 3 hours at 37°C under 5% CO2, the labeling medium was removed, followed by fixation and final incubation with anti-BrdU-POD solution. The absorbance of the samples was measured using an ELISA reader (Promega) at 450 nm, with the absorbance at 690 nm as reference.

### Statistical analysis

Statistical analyses were performed using SPSS V.14.0 software (SPSS Inc. Chicago, IL, USA). The median H-score of immunohistochemistry for MCM2 and MCM10 was used as the cutoff to dichotomize the study cohorts into high and low expression groups. Pearson's *x^2^* test was used to compare MCM2 and MCM10 expression and various clinicopathological features. The end points analyzed were disease-specific survival (DSS) and metastasis-free survival (MeFS). DSS and MeFS were calculated from the starting date of curative surgery to the date of an event development. Univariate and multivariate analyses were performed using the Kaplan-Meier method with the log-rank test and the Cox proportional hazards model. Student's t-test was used to analyze quantitative RT-PCR and functional assays for cell line samples. Statistical significance was set at P < 0.05.

## SUPPLEMENTARY MATERIALS TABLES


