# Supplementary material for: MCM10 overexpression implicates adverse prognosis in urothelial carcinoma
Source: Oncotarget. 2016 Oct 21;7(47):77777–92. doi: 10.18632/oncotarget.12795 (PMC5363620; doi:10.18632/oncotarget.12795)
Supplement: Supplementary file 1 [file oncotarget-07-77777-s001.pdf]

MCM10 overexpression implicates adverse prognosis in urothelial carcinoma

SUPPLEMENTARY TABLES

Supplementary Table S1: Correlations between MCM10 and MCM2 Expression and other important clinicopathological parameters in urothelial carcinomas

		Urothelial Cancer of upper urinary tract							Urothelial cancer of urinary bladder						
**Parameter**	**Category**	**Case No.**	**MCM10 Exp.**		**p-value**	**MCM2 Exp.**		**p-value**	**Case No.**	**MCM10 Exp.**		**p-value**	**MCM2 Exp.**		**p-value**
			**Low**	**High**		**Low**	**High**			**Low**	**High**		**Low**	**High**	
**Primary tumor (T)**	Ta-T1	16	13	3	**0.004***	12	4	**0.048***		11	1	**0.004***	10	2	**0.005***
	T1	9	5	4		4	5			2	5		5	2	
	T2-T4	25	7	18		9	16			12	19		10	21	
**Nodal metastasis (N)**	Negative (N0)	46	24	22	0.297	24	22	0.297		22	21	0.684	22	21	0.684
	Positive (N1-N2)	4	1	3		1	3			3	4		3	4	

* Statistically significant.

Supplementary Table S2: Univariate analyses for Disease-specific and Metastasis-free Survivals in Upper urinary tract urothelial carcinoma

Parameter	Category	Case No.	Urothelial Cancer of upper urinary tract				Urothelial cancer of urinary bladder			
			Disease-specific survival		Metastasis-free survival		Disease-specific survival		Metastasis-free survival	
			No. of event	p-value	No. of event	p-value	No. of event	p-value	No. of event	p-value
**MCM10 Exp.**	Low	25	3	**0.0156***	4	**0.0178***	2	**0.0059***	3	**0.0115***
	High	25	12		12		7		8	
**MCM2 Exp.**	Low	25	4	**0.0409***	6	0.2111	2	**0.0466**	4	0.2471
	High	25	11		10		7		7	

* Statistically significant.

Supplementary Table S3: Multivariate analyses for Disease-specific and Metastasis-free Survivals in Upper urinary tract urothelial carcinoma

Parameter	Urothelial Cancer of upper urinary tract				Urothelial cancer of urinary bladder			
	Disease-specific survival		Metastasis-free survival		Disease-specific survival		Metastasis-free survival	
	R.R	p-value	R.R	p-value	R.R	p-value	R.R	p-value
**MCM10 Exp.**	**3.632**	**0.048***	**3.338**	**0.041***	**5.131**	**0.048***	**4.440**	**0.037***
**MCM2 Exp.**	2.574	0.108	1.502	0.441	2.761	0.221	1.288	0.703

* Statistically significant.
